# Supplementary material for: Is body fat mass associated with worse gross motor skills in preschoolers? An exploratory study
Source: PLoS One. 2022 Mar 9;17(3):e0264182. doi: 10.1371/journal.pone.0264182 (PMC8906635; doi:10.1371/journal.pone.0264182)
Supplement: S1 File — (DOCX) [file pone.0264182.s002.docx]

Regression between independent variables and Standard Score Object Control (N=48).

|  | Simple linear regression  Locomotor subset standard score | | | | |
| --- | --- | --- | --- | --- | --- |
| Variable | ẞ | B | 95% CI | *p-value* | R^2^ |
| Classification of PA | 0.214 | 0.960 | -0.35-(2.27) | 0.147 | 0.025 |
| Sex | -0.163 | -0.735 | -2.05-(0.58) | 0.267 | 0.006 |
| Age (years) | 0.025 | 0.056 | -0.08-(0.13) | 0.659 | -0.017 |
| Economic status | 0.123 | 0.058 | -0.50-(0.74) | 0.695 | -0.018 |
| EC-HOME | 0.038 | 0.093 | -0.08-(0.15) | 0.530 | -0.013 |
| Quality of the school environment (ERCS) | 0.720 | 0.091 | -1.60-(3.04) | 0.536 | -0.013 |
| Body fat mass | -0.081 | -0.147 | -0.242-(-0.080) | 0.318 | 0.000 |

Note: ẞ= standard regression coefficient; B = non-standard regression coefficient; 95% CI = 95% confidence interval; estimate of the increase or decrease of the dependent variable for each increase of one unit of the independent variable; p = statistical significance; R^2^ = coefficient of determination. PA: physical activity. EC-HOME: Early Childhood Home Observation for Measurement of the Environment. ECERS: Early Childhood Environment Rating Scales.
