# Supplementary material for: Is body fat mass associated with worse gross motor skills in preschoolers? An exploratory study
Source: PLoS One. 2022 Mar 9;17(3):e0264182. doi: 10.1371/journal.pone.0264182 (PMC8906635; doi:10.1371/journal.pone.0264182)
Supplement: Attachment — Submitted filename: Review Ms PONE.pdf [file pone.0264182.s003.pdf]

Review Ms PONE-D-21-16031

Is body fat mass associated with worse gross motor skills in preschoolers? An exploratory study.

The authors compared motor competence in a relatively small sample of overweight/obese and eutrophic children (5 yrs of age), thereby taking a series of secondary variables (PA, sex, gender, age, SES, etc…) into account. While the underlying rationale of the study is to be applauded, and the authors collect an impressive set of variables with state-of-the-art assessment instruments (great effort, much appreciated!!!), the writing of the paper itself is problematic and unacceptable for publication in this form. I had the impression that this paper was written under severe time pressure, resulting in a kind of 'quick & dirty job'.

I hope that my comments below are helpful for the authors to rework this material that does have publication potential, but not in its current form.

Language:

This manuscript needs a thorough revision by a native English speaker or by a professional familiar with the writing of scientific reports.

Abstract:

- Use the same tense during the abstract
- 'quantitative': how to be interpreted here? Should be quantitatively?
- Sentence with 'but similar skills'… check grammar
- Globale language revision is mandatory

Introduction:

- The introduction is too short and superficial to allow the development of a solid rationale for this study. Specifically with respect to the weight/motor skill relationship, the authors can start from the abundance of literature in children from 5 years and older, describe what we know already in more detail (point to the urgency of the problem), and then move on tot he part in which the paucity of similar studies in younger children is described. This approach will also help to formulate specific hypotheses at the end of the introduction. You may want to consults the publications of Eva D'Hondt / Mireille Augustijnen between 2008-2010 for that purpose.
- I applaud the inclusion of the impressive set of secondary variables, but the rationale on why to include one variabele (and not other ones) is lacking. Suggestion to shortly describe for each of the variables whether they might have a positive or a negative effect on motor competence. Again, this will help building a solid set of hypotheses.
- Describing the strength of the study does not belong at the end of the introduction

Methods:

- More information on the recruitment procedure is needed. How many parents were contacted for each group and how many agreed participation of their child?
- I assume you mean that you excluded participants that were subject to an infectious process at the time of the study (not …had been subject..)?
- I agree that the sample size is large enough for the straightforward comparison between groups, but did the authors also reckon with the extended set of secondary variables in their power analysis?
- How is age-specific BMI age specific as you just calculate weight/m²? Please be more specific.
- Please provide reliability/validity information on each test instrument
- ACtigraph: more details are needed. Were the 3 days randomly chosen? Weekdays and/or weekend days? Please first describe the instrument, then how it is positioned, and then information on the analysis. Not clear how you can capture heart rate with a hip-mounted device. How is 'the child's mean time' calculated?
- TGMD-2: 12 motor skills divided into two categories (not subtests)
- Data are presented as mean or median, as appropriate. I assume you mean 'depending on the outcome of the SW and L test. See comment under results.
- The statistics section lacks structure, and this reflects in the Tables. It is very hard to figure out what test was use for what variable from the tables. I am not convinced that it is necessary to strictly adhere to the results of the SW and L test to decide on the statistical approach for each variable independently given the –in my opinion- sufficiently large number of participants per group.

Results:

- Table 1: please provide the units for each variable (eg under 'sex' it is N, with the proportion in brackets; is amount of body mass in kg or a %; etc.)
- Subscript 'a' is floating in the table, where does it belong to?
- I would say that children this age 'attend' school rather than 'study'
- Of course it makes sense that body mass differed between groups, given that children were selected on being overweight.
- Table 2: mixing between parametric and non-parametric stats in subscores of the same testbattery is confusing
- Please provide also the results of the OC analyses. Not finding significant differences is as important as reporting the significant ones!

Discussion:

- The last paragraph of the results is confusing: first you mention the MLR adjusted for confounding factors. You end the paragraph with …'Finally, control variables were inserted in the model….' Isn't that twice the same?
- The participants were selected from public schools in the same municipality, which might explain the absence of differences in the secondary variables? Discuss where this group is to be situated compared to rest of the Brazilian children?
- The results could be better framed within the knowledge on children that are slightly older. For example, several studies did find differences in fine motor skills in young obese children (I think D'Hondt et al in Neuroscience Letters or APAQ somewhere in 2008 or 2009). Might be worthwile discussing in a broader context.

- On p 14, I cannot understand why the concept of ‘body image’ suddenly emerges in the discussion. Difficult sentence to grab, please clarify.
- You may want to consult the recent meta-analysis by Barnett et al in Sports Medicine (2021), overviewing among others the longitudinal relationship / causality between weight status and MC (and the reverse).
- Near the end of the discussion and practical implications: suggestion to stick tot he findings of this study. This was a cross-sectional study, so the conclusion that excess body fat mass influences competence in locomotor skills should be toned down a bit.
- The practical implications do not relate to the findings of this study at all, please revise.
