# Supplementary material for: Is body fat mass associated with worse gross motor skills in preschoolers? An exploratory study
Source: PLoS One. 2022 Mar 9;17(3):e0264182. doi: 10.1371/journal.pone.0264182 (PMC8906635; doi:10.1371/journal.pone.0264182)
Supplement: Attachment — Submitted filename: Response to reviews.docx [file pone.0264182.s005.docx]

Ref: PONE-D-21-16031

Title: Is body fat mass associated with worse gross motor skills in preschoolers? An exploratory study

PLOS ONE

Dear Daniel V. Chagas

Academic Editor

PLOS ONE

We appreciate the reviewer’s comments. They were important to improve the quality of our manuscript. See below the point-to-point answers.

PONE-D-21-16031

1) Overall, the manuscript was amended addressing all issues raised by reviewers, including my comments as reviewer in the first round of revision. However, I would like to suggest some papers to be considered in the manuscript, especially in the Introduction and Discussion. These articles are: Hall et al. 2018 (doi:10.3390/jfmk3040057); Wood et al. 2022 (doi: 10.1089/chi.2021.0026); Henrique et al. 2019 (doi.org/10.1002/ajhb.23364)
A) Hall et al. 2018 (doi:10.3390/jfmk3040057

Answer: We appreciate the suggestion. We inserted the reference in the introduction (line 60) “healthy behaviors that may influence physical activity (PA) practices throughout life [10, 11]”.

B) Wood et al. 2022 (doi: 10.1089/chi.2021.0026)

Answer: We appreciate the suggestion. We inserted the reference in the introduction (see lines 67-68: “Although previous work has shown that fat mass was not related to any motor skill measure [13]” and lines 322-323) “Although previous work has shown that fat mass was not related to any motor skill measure [13]”.

C) Henrique et al. 2019 (doi.org/10.1002/ajhb.23364)

We appreciate the suggestion. We inserted the reference in the introduction (see line 69: including a work with Brazilian preschoolers from the Northeast region of Brazil [15]), and in the discussion (see lines 329-330: In this sense, we emphasize that this relationship can be bidirectional from a preschool age [15]). Of note, the suggested article was already listed in the references (see reference 15).

Thank you for contacting us and we are at your disposal for any questions you may have.

Sincerely,

Juliana Nobre, PhD

Universidade Federal dos Vales do Jequitinhonha e Mucuri, Brazil
